# What Is the Relationship Between Sensory Attributes Identified Using CATA (Check-All-That-Apply) Questionnaire and Consumer Acceptance of Cookies Using Plant-Based Oils?

**DOI:** 10.3390/foods13223593

**Published:** 2024-11-10

**Authors:** Juyoun Lee, Seyoung Ju

**Affiliations:** 1 Department of Food and Nutrition, Duksung Women’s University, 33 Samyang-ro, Seoul 01369, Republic of Korea; foodinstitute@hanmail.net; 2 Department of Food & Nutrition, College of Biomedical and Health Science, Konkuk University, Chungju 27478, Republic of Korea

**Keywords:** plant-based cookies, consumer acceptance, Check-All-That-Apply (CATA), correspondence analysis (CA), heatmap, multiple factor analysis (MFA)

## Abstract

The purpose of this study was to investigate sensory attributes identified using a CATA questionnaire and consumer acceptance of cookies using plant-based oils and to determine which sensory attributes influence and improve purchase intention of cookies using plant-based oils. Frequency analysis, one-way analysis of variance, correspondence analysis, Pearson correlation analysis, and multiple factor analysis were used for statistical evaluation. Six cookie samples, five with types of vegetable oil (canola, brown rice, coconut, soybean, olive) and one with butter, were evaluated by 112 consumers. Of the 28 sensory attributes, the 11 sensory terms of milk, savory, sweet aroma/odor, sweet, roasted grain, milk taste/flavor, hardness, “crispy”, “stuffy”, roughness, and “crumble” were significantly different among samples (*p* < 0.001). In the consumer acceptance and purchase intention tests, participants evaluated 12 attributes, namely, overall liking, appearance, color, aroma/smell, overall taste, savory flavor, flavor, sweet taste, aftertaste, texture, “smooth”, “crispy”, “try again”, recommendations, and familiarity, on a 9-point structured hedonic scale. All attributes except “smooth” were significantly different among the six cookie samples tested (*p* < 0.001). Samples with coconut oil and butter had the highest consumer acceptance. In particular, in a correspondence analysis, the samples with butter and coconut oil positioned in the positive direction (+) of Dimension (Component) 1 were closely related with mouthfeel, sweet aroma, sweet taste, “crispy”, “try again”, recommendation, familiarity, and overall liking. Our results suggest that cookies with coconut oil could be a substitute for animal-fat-based cookies with butter. We hope that our research will provide fundamental data for the development of vegan bakery products that meet the needs of consumers and food companies.

## 1. Introduction

The global market size of cookies is expected to grow at a current annual growth rate of 5.3% from 2019 to 2025. Due to consumer demands and various social issues such as obesity and health, the manufacturing and sales of oatmeal, vegan, sugar-free, and gluten-free cookies are growing significantly [1]. In addition, there has been increasing interest in the development and research of sustainable alternatives to animal-based foods because of ethical and environmental concerns [2]. Cookies are a popular bakery product that can be enjoyed without any burden. Cookies are generally formulated with wheat flour, butter or shortening, sugar, eggs, salt, and other ingredients. Butter or shortening is mainly saturated fatty acids that are solid state at room temperature, and this solid form of butter is important for determining the texture and taste of cookies [3]. Specifically, fat (butter) in cookies plays a very important and essential role in determining plasticity, creaminess, foaming, and emulsifying properties, contributing to the soft, crunchy texture and taste of cookies. However, the excessive consumption of these saturated and trans fatty acids (butter, shortening, and margarine) is closely related to many health problems such as obesity, cardiovascular diseases, and dyslipidemia [4,5,6]. According to the American Heart Association for preventing cardiovascular diseases, the consumption of vegetables and fruit, whole grains, seeds and nuts, and vegetable oils is recommended [5]. In this sense, many researchers and manufacturers have tried to replace fats with alternatives such as vegetable oil or fat substitutes that are not high in saturated and trans fatty acids as ingredients of bakery products [7,8,9]. Omega-3 polyunsaturated fatty acids, found in vegetable oils, are known to reduce platelet coagulation and cardiovascular diseases [10]. Moreover, recent research has shown that omega-3 polyunsaturated fatty acids are effective in preventing depression by helping with brain activity [11,12].

According to the International Vegetarian Union, there are approximately 180 million vegetarians in the world, and the vegetarian population in Korea by 2022 is estimated to have reached 1.5–2 million, or 3–4% of the total population [13]. A plant-based diet must obtain nutritional adequacy from a variety of plant foods, abounding in complex carbohydrates, proteins, omega-3 fatty acids, vitamins, minerals, and fiber. These nutrients are abundant in grains, legumes, seeds, nuts, vegetables, and fruits. Among these plant groups, protein-rich plant foods (legumes) and vegetable oils (seeds and nuts) play very important roles in plant-based diets [6]. Social awareness and attitudes toward vegetarianism continue to change in a positive direction, and in addition, the number of plant-based foods is increasing due to innovations in the food-tech industry. This change has led to much research being conducted on products that use plant-based ingredients in bakery products that currently mainly use animal-based products [2,3,14,15,16,17]. Milk or butter, the main ingredients of bakery products, can be replaced with plant-based milk (soy or oat) and vegetable oil. Previous related studies on developing plant-based bakery products mainly studied the quality characteristics using olive oil [14,15], grape seed oil [16,17], coconut oil [2,14], soybean oil [17,18], chia oil [19], and other seeds and nuts [3]. In addition, studies on the quality characteristics of hamburger patties [20] and chocolate [21] using vegetable oils have been reported. Furthermore, studies on the use of microencapsulated vegetable oil powder, oleogel, or food-grade bigels to develop vegan cookies have been presented [22,23,24]. These studies are expected to lead to healthier and more nutritious bakery products by mitigating oxidation due to the high unsaturation of vegetable oils.

In this study, we conducted an investigation of sensory attributes by CATA questionnaire and consumer acceptance of cookies with five plant-based oils, to determine which sensory characteristics influence purchase intention and to improve purchase intention of cookies using plant-based oils.

## 2. Materials and Methods

### 2.1. Materials

The materials used in the experiment were as followed: wheat flour (Sajo, Dangjin, Chungcheongnam-do, Republic of Korea), almond powder (SOSA, Barcelona Spain), sugar powder (breadgarden, Seongnam, Gyeonggi-do, Republic of Korea), corn starch (Hagawon food, Nonsan, Chungcheongnam-do, Republic of Korea), canola oil (Haepyo, Incheon, Republic of Korea), brown rice oil (Rizi, Bangkok, Thailand), coconut oil (Farmers Origin, Colombo, Sri Lanka), soybean oil (Haepyo, Incheon, Republic of Korea), olive oil (Amoliva Pomace, Valencia, Spain), and butter (Elvir Elle & Vire, Paris, France). The five types of plant-based oils used in this study were selected based on the ranking of the online distributor most used by Koreans.

### 2.2. Preparation of Samples

Six samples of cookies were prepared by selecting five types of plant-based oil (canola, brown rice, coconut, soybean, olive) and butter (control sample). The compositions of the cookies are presented in Table 1. The composition of the cookies was formulated through an analysis of previous studies, several preliminary experiments, and preliminary consumer tests [2,3,6,7]. The method of cookie preparation was as follows. First, after sieving the powders, all measured ingredients were placed in a food processor (KitchenAid, Benton Harbor, MI, USA) and thoroughly mixed for three minutes. The resulting dough was wrapped in parchment paper and left to rest in the refrigerator (2 ± 1 °C) for two hours. Then, the dough was rolled out to a thickness of 5mm and shaped into a square using a cookie cutter, before finally being placed on an oven pan lined with parchment paper and baked at 160 °C for about 12 min. The cookies were wrapped in plastic bags after cooling.

### 2.3. Consumer Test—Consumer Acceptance and CATA

A total of 112 consumers (females: 54, males: 58, age: 20–30 years) participated in the consumer acceptance test. Participants who were allergic to any ingredient of the sample cookies were excluded from the consumer test. This study was approved by the Institutional Review Board of Konkuk University (7001355-202408-HR-831). All samples were served on a white paper dish (10 cm in diameter) with drinking water for participants to cleanse their mouths between samples to prevent residual effects. Samples coded with random three-digit numbers were provided. A three-digit random number based on the Williams Latin Square Design was used in the samples to minimize errors depending on the order of presentation [25].

The test consisted of two parts; the first part measured consumer acceptance for 12 attributes (overall liking, appearance, color, aroma/smell, overall taste, savory flavor, flavor, sweet taste, aftertaste, texture, “smooth”, ”crispy”) on a 9-point structured hedonic scale (1 = disliked strongly, 5 = neither liked nor disliked, 9 = liked strongly) and 3 attributes (try again, recommendation, familiarity) on a 9-point scale ranging from 1 (strongly disagree) to 9 (strongly agree). The second assessment involved CATA questions to check the attributes that consumers selected appropriate to describe the sample cookies. The attributes for the CATA questions included appearance (color, glossy, uniformity), aroma/odor (milk, savory, rancid odor, sweet, off-odor), taste/flavor (savory, greasy, sweet, salty, bitter, oily, nutty, raw flavor, roasted flour, roasted grain, milk, off-flavor), texture/mouthfeel (hardness, crispy, stuffy, oily, roughness, residual sensation, “crumble”, “smooth”) were determined from previous studies [3,15,17,18,19,26].

### 2.4. Statistical Analysis

Frequency Analysis was performed to calculate the frequency and percent ratio of consumers’ answers to the CATA (Check-All-That-Apply) questionnaire. Chi-square tests and, when the occurrence of a frequency of less than 5 for each item was more than 20%, Fisher’s exact tests were conducted to confirm significance between samples. Correspondence analysis (CA) was performed for visualization of the relationship between six samples. One-way analysis of variance (ANOVA) of sensory attributes was applied to compare consumer acceptability and purchase attention between the samples, followed by Duncan’s multiple range post-hoc test when a significant difference was present at α = 0.05. In addition, multiple factor analysis was performed on the average of preference and purchase intention for each sample and the ratio of sensory attributes and visually analyzed correlation between sensory attributes and liking drivers. The correlation between liking drivers was presented as a heatmap based on Pearson’s correlation coefficient. ANOVA, frequency analysis, chi-square test, and Fisher’s exact test were conducted using SPSS Statistics Ver. 18.0 (SPSS Inc., Chicago, IL, USA), and multiple factor analysis and Pearson correlation analysis were performed using R Statistical System 4.1.2 (R Development Core Team, 2011).

## 3. Results and Discussion

### 3.1. General Characteristics

A total of 112 consumers were recruited to test consumer acceptance and answer CATA questions for cookies with plant-based oils. Table 2 presents the general characteristics of the participants in the consumer test. The gender ratio was 58% men and 42% women, of which 87.5% were single. In terms of frequency of cookie intake, those who consumed cookies 2–3 times per month were the most numerous at 27.7% of participants, followed by once per month at 17.9%. The percentage of participants who consumed cookies 1–2 times and 3 or more times per week was quite high at 17.9%. More than 50% of the participants used bakery stores when purchasing cookies. A total of 65.2% responded that they had experienced vegan food. These results show that cookies are a popular bakery product and it was also found that vegan food has received increased awareness and interest from consumers.

### 3.2. Frequency of Sensory Characteristics and Correspondence Analysis of Cookie Samples Identified Using CATA

Table 3 shows the frequency with which 28 sensory characteristics were mentioned by participants for cookie samples prepared with plant-based oil and a standard prepared with butter obtained using CATA questions. The definition of CATA attributes from cookies using plant-based oils in Table S1. The frequency of 11 sensory terms of milk, savory, and sweet aroma/odor, sweet, roasted grain, and milk taste/flavor, hardness, “crispy”, “stuffy”, roughness, and “crumble” (texture/mouthfeel) varied significantly among samples (*p* < 0.001). The sensory term of savory taste had a higher frequency than other sensory terms (70.5–84.8%), but there was no significant difference between samples. This result shows that a savory taste could be assumed to be the common characteristic of these cookies. Milk odor had a significant difference between samples, the sample with butter appeared to have the highest frequency among samples while the sample with olive oil showed the lowest frequency among them. Conversely, in terms of roasted grain taste, the sample with olive oil had the highest frequency, while the sample with butter showed the lowest frequency among samples.

**Figure 1:**
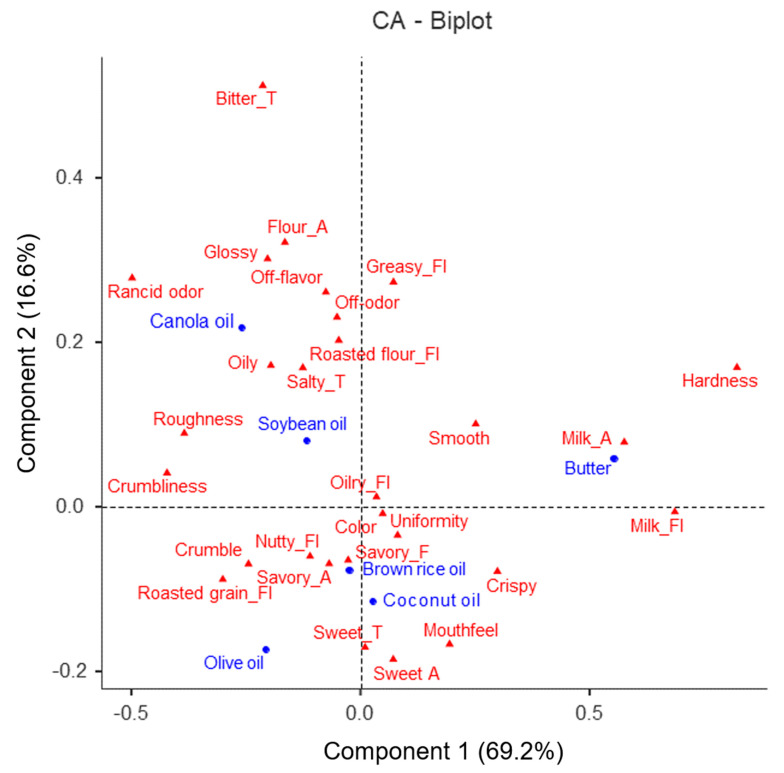
Correspondence analysis (CA) biplot of six cookie samples using 28 sensory attributes identified using CATA. Dim is an abbreviation for dimension.

Figure 1 presents the correspondence analysis (CA), which visually shows the correlation ship between the 28 sensory characteristics identified using CATA for the six cookie samples prepared with plant-based oil or butter. The CA plot explained a total variance of 85.8%, which accounted for the combination of 69.2% of the variance with Component 1 and 16.6% with Component 2. Samples with butter positioned in the positive (+) direction of Component 1 were closely related with milk aroma, while samples with coconut oil were closely located with sweet taste and mouthfeel. Since butter is a fat made from milk, it should naturally have a closer relationship with a milk flavor than the samples prepared with plant-based oils. Choi and Jung’s study [9] also found that scones prepared with coconut oil had the highest flavor compared to scones prepared with other oils and fats such as butter, fresh cream, and olive oil. Coconut oil is a vegetable oil, but its composition of fat is similar to that of animal fat (butter), which has a high content of saturated fatty acids. Therefore, cookies with coconut oil had a better mouthfeel than those of other vegetable oils.

Samples with canola oil were positioned in the negative direction (-) of Component 1 and the positive direction (+) of Component 2, affecting attributes of disparate aroma, disparate flavor, and rancid odor. Samples with brown rice oil located in the negative (-) direction of Component 2 were closely correlated with attributes such as savory flavor, savory aroma, nutty flavor, roasted grain flavor, and “crumble”.

### 3.3. Consumer Acceptance

Table 4 presents the results of the consumer acceptance test for the six cookie samples. The evaluation of consumers was conducted based on the attributes of overall liking, appearance, color, aroma/smell, overall taste, savory flavor, flavor, sweet taste, aftertaste, texture, “smooth”, and “crispy” using a 9-point hedonic scale. The test result shows all attributes except “smooth” were significantly different among the six cookie samples (*p* < 0.001). Samples with coconut oil and butter had the highest consumer acceptance. Samples with coconut oil were rated the highest in terms of acceptance of overall liking, aroma/smell, overall taste, flavor, sweet taste, aftertaste, and “smooth”. Samples with olive oil showed the highest score for savory flavor among samples and had a high score in overall taste. This result shows that consumers prefer cookies with coconut oil and butter because the texture and mouthfeel of bakery products are quite affected by the type and content of fat [9]. This result shows that the cookies with coconut oil and butter had the highest overall liking because coconut oil has a high content of saturated fatty acids similar to that of butter. In our study result, cookies with coconut oil had the highest overall liking, followed by cookies with butter and those with olive oil, while cookies with canola oil had the lowest score of overall liking among the six sample cookies. Another research reported similar results to our study. Choi and Jung [9] investigated the effects that the use of butter, fresh cream, olive oil, and coconut oil had on the quality characteristics of rice scones. They found that scones with coconut oil had the highest overall acceptability. Conversely, Shin et al. [12] studied the quality characteristics of iced pumpkin cookies made with butter, olive oil, soybean oil, and grape seed oil. Their result showed that cookies made with grape seed oil had the highest overall liking, followed by cookies with olive oil, and cookies with soybean oil, while cookies with butter had the lowest overall liking among them.

### 3.4. Pearson Correlation Coefficient Heatmap of Consumer Acceptance

**Figure 2:**
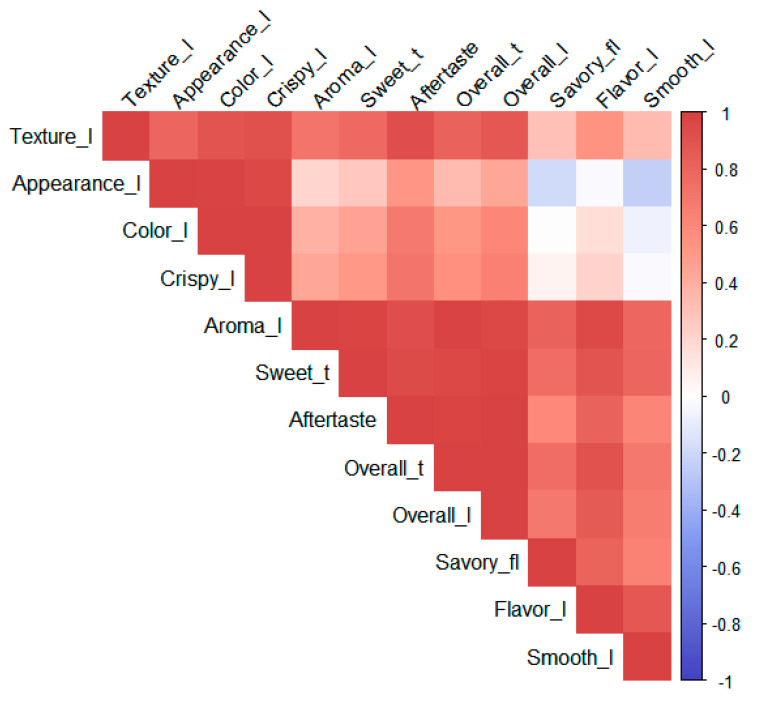
Pearson correlation coefficient heatmap regarding the consumer acceptance of cookie samples.

Pearson correlation analysis was performed to identify the correlation between sensory attributes of samples by consumer acceptance test. The results are visually represented as a heatmap in Figure 2. A heatmap is a data visualization technique that shows relationships between two variables and represents the magnitude of individual values as color intensity [27]. The attributes that showed a high positive (+) correlation with overall liking were overall taste (r = 0.99, *p* < 0.001), aroma (r = 0.96, *p* < 0.001), sweet taste (r = 0.97, *p* < 0.001), aftertaste (r = 0.99, *p* < 0.001), texture (r = 0.87, *p* < 0.001), and flavor (r = 0.86, *p* < 0.001). This result demonstrates that the sensory attributes of aroma, sweet taste, aftertaste, texture, and flavor may affect the selection and purchase of cookies.

### 3.5. Purchase Intention

Table 5 presents the results of evaluating the purchase intention of cookies using plant-based oils by consumer panels. The purchase intention of consumers was investigated using attributes such as “try again”, recommendation, and familiarity. All attributes were significantly different among samples (*p* < 0.001). Samples with coconut oil showed the highest purchase intention for all three attributes, while samples with canola oil had the lowest scores. Since purchase intention is closely related to consumer preference, cookies with coconut oil, which marked the highest score in consumer acceptance, also had the highest scores of purchase intention. Samples with olive oil had the highest scores in purchase intention among cookies with vegetable oils except those with coconut oil. When purchasing or selecting cookies, sensory attributes such as texture and mouthfeel are important affecting factors. The presence of high saturated fatty acids in butter or coconut oil in cookies could deliver the desired texture and mouthfeel [8]. Therefore, it appears that consumers prefer these two types of cookies and have high purchase intention, recommendation, and familiarity.

### 3.6. Multiple Factor Analysis (MFA) by Sensory Characteristics, Consumer Acceptance, and Purchase Attention

**Figure 3:**
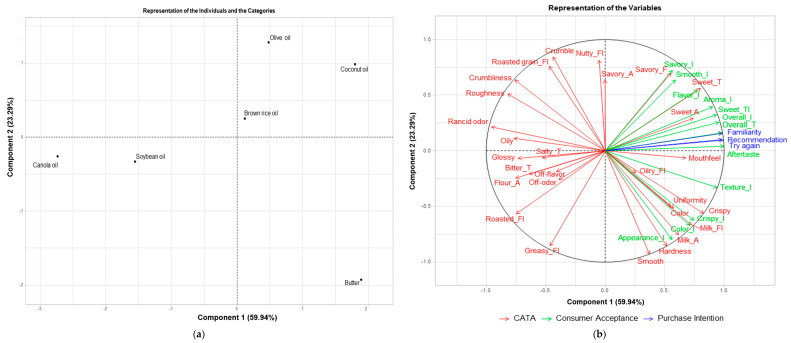
Results of Multiple Factor Analysis (MFA) by sensory characteristics, consumer acceptance, and purchase attention of six cookie samples; plots for samples (**a**) and sensory characteristics identified using CATA, consumer acceptance, and purchase intention (**b**).

In Figure 3, MFA is used to visually present the correlation between sensory characteristics identified using CATA, consumer acceptance, and purchase intention. The plot shows a total variance of 83.23%, representing a combination of 59.94% from Component 1 and 23.29% from Component 2. Samples with butter and coconut oil positioned in the positive direction (+) of Component 1 were closely related to mouthfeel, sweet aroma, sweet taste, “crispy”, “try again”, recommendation, familiarity, and overall liking. This result demonstrates that there appears to be a close relationship between sensory attributes such as taste, mouthfeel, texture, and aroma because butter and coconut oil contain high quantities of saturated fatty acids. Coconut oil is 90% saturated fats and 9% unsaturated fats. However, its composition of saturated fats is different from that of animal fats. More than 50% of coconut oil consists of medium-chain fatty acids such as lauric acid (C 12) and myristic acid (C 14). It is known that coconut oil is directly absorbed in the intestine to produce energy and is effective against heart disease and chronic health problems [28,29,30,31].Conversely, samples with soybean oil and canola oil located in the negative direction (-) of Component 1 had a close relationship with off-odor, rancid odor, off-flavor, and raw flour flavor. Samples with olive oil positioned in the positive direction (+) of Component 2 were correlated with savory aroma and nutty flavor. 

## 4. Conclusions

Cookies using plant-based oils (canola, brown rice, coconut, soybean, olive oil) were prepared to investigate consumer perception of sensory attributes identified using CATA and consumer acceptance. A total of 11 sensory terms identified by conducting a 28-term CATA-based analysis, namely, milk, savory, and sweet aroma/odor, sweet, roasted grain, and milk taste/flavor, hardness, “crispy”, “stuffy”, roughness, and “crumble”, were significantly different among the samples (*p* < 0.001). The results of a consumer acceptance test showed that, out of 12 attributes, all except “smooth” were significantly different among the six cookie samples (*p* < 0.001). Samples with coconut oil and butter had the highest consumer acceptance. Samples with coconut oil were rated the highest in acceptance of overall liking, aroma/smell, overall taste, flavor, sweet taste, aftertaste, “smooth”, and purchase intention. In a multiple factor analysis, samples with coconut oil and butter positioned in the positive direction (+) of Component 1 were closely related with mouthfeel, sweet aroma, sweet taste, “crispy”, “try again”, recommendation, familiarity, and overall liking. Excessive consumption of animal oils such as butter can cause heart disease, while coconut oil contains a high proportion of MCFAs among its saturated fatty acids, so has a low risk of disease. The presence of saturated fatty acids is an important factor in sensory characteristics such as texture and mouthfeel that determine the quality of bakery products. Since coconut oil is similar to animal fat, it can be used as a good substitute for butter. Due to environmental changes and food technological advancements, the consumption of and interest in plant-based products are increasing. Plant-based foods continue to be developed and commercialized; however, compared to animal-based foods, although they have a positive effect on health, they often fall short in terms of consumers’ taste needs. Modern consumer needs include food that is not only healthy, ethical, and environmentally friendly but also palatable, so the food industry and researchers are investigating a variety of new functional alternatives to animal-based foods, and plant-based foods meet these new demands [32]. We hope that the results of our research will be used as basic data for making bakery products using plant-based oils, leading to the development of products that meet the needs of consumers and the expectations of food manufacturers. 

## Figures and Tables

**Table 1:** Composition of cookies using plant-based oils.

Ingredient(g)	Types of Oil					
	Butter (470)	Canola Oil (918)	Brown Rice Oil (613)	Coconut Oil (762)	Soybean Oil (396)	Olive Oil (284)
Wheat Flour	30	30	30	30	30	30
Almond powder	20	20	20	20	20	20
Sugar powder	20	20	20	20	20	20
Cornstarch	3	3	3	3	3	3
Butter	25	-	-	-	-	-
Canola oil	-	25	-	-	-	-
Brown rice oil	-	-	25	-	-	-
Coconut oil	-	-	-	25	-	-
Soybean oil	-	-	-	-	25	-
Olive oil	-	-	-	-	-	25
	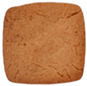	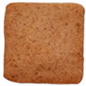	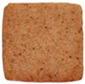	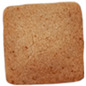	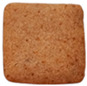	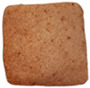

**Table 2:** General Characteristics of Survey Subjects (*n* = 112).

Variables		*n*	%
Gender	Male	58	51.8
	Female	54	48.2
Age (Mean, Standard Deviation)		25.0	7.6
Marital status	Single	98	87.5
	Married	14	12.5
Family number (Mean, Standard Deviation)		2.7	1.4
Place of residence	Apartment	51	45.5
	Studio flat	41	36.6
	Dormitory	8	7.1
	Multi-unit house	7	6.3
	Detached house	4	3.6
	Other	1	0.9
Household income (Unit: 10,000 Won)	<100	14	12.5
	100–300	19	17.0
	300–500	15	13.4
	500–700	27	24.1
	≥700	37	33.0
Frequency of cookie intake	≥3/Week	5	4.5
	1–2/Week	15	13.4
	2–3/Month	31	27.7
	1/Month	20	17.9
	1/2–3Months	9	8.0
	<3/Months~≥2/Year	16	14.3
	Seldom(<1/Year)	16	14.3
How to Buy Cookies	Department store	7	6.3
	Wholesale Market	12	10.7
	Supermarket	13	11.6
	Bakery	63	56.3
	Homemade	8	7.1
	Online	5	4.5
	Others	4	3.6
Experience of eating vegan food	Yes	73	65.2
	No	39	34.8

**Table 3:** Results for sensory attributes of six cookie samples (n = 112).

Sample		Butter (470)		Canola Oil (918)		Brown rice Oil (613)		Coconut Oil (762)		Soybean Oil (396)		Olive Oil (284)		*p*-Value ^(1)^
		n	%	n	%	n	%	n	%	n	%	n	%	
Appearance	Color	69	61.6	55	49.1	55	49.1	65	58.0	64	57.1	57	50.9	0.272
	Glossy	8	7.1	22	19.6	11	9.8	13	11.6	13	11.6	6	5.4	0.014
	Uniformity	70	62.5	51	45.5	63	56.3	50	44.6	48	42.9	58	51.8	0.021
Aroma/Odor	Milk	69	61.6	21	18.8	24	21.4	32	28.6	17	15.2	15	13.4	<0.001
	Savory	78	69.6	82	73.2	85	75.9	80	71.4	82	73.2	104	92.9	<0.001
	Rancid odor	1	0.9	10	8.9	5	4.5	2	1.8	7	6.3	6	5.4	0.049 ^(2)^
	Sweet	43	38.4	28	25.0	53	47.3	64	57.1	35	31.3	37	33.0	<0.001
	Off-odor	14	12.5	18	16.1	7	6.3	18	16.1	25	22.3	8	7.1	0.003
Taste/Flavor	Savory	84	75.0	79	70.5	86	76.8	92	82.1	87	77.7	95	84.8	0.134
	Greasy	29	25.9	28	25.0	20	17.9	15	13.4	30	26.8	12	10.7	0.005
	Sweet	60	53.6	47	42.0	68	60.7	80	71.4	51	45.5	69	61.6	<0.001
	Salty	7	6.3	12	10.7	9	8.0	12	10.7	16	14.3	5	4.5	0.129 ^(2)^
	Bitter	4	3.6	12	10.7	4	3.6	4	3.6	3	2.7	2	1.8	0.015 ^(2)^
	Oily	26	23.2	23	20.5	22	19.6	31	27.7	28	25.0	19	17.0	0.427
	Nutty	36	32.1	52	46.4	48	42.9	58	51.8	41	36.6	50	44.6	0.044
	Raw flour	14	12.5	30	26.8	15	13.4	10	8.9	13	11.6	12	10.7	0.002
	Roasted flour	25	22.3	31	27.7	27	24.1	16	14.3	33	29.5	17	15.2	0.025
	Roasted grain	26	23.2	66	58.9	52	46.4	52	46.4	63	56.3	87	77.7	<0.001
	Milk	58	51.8	9	8.0	18	16.1	31	27.7	15	13.4	10	8.9	<0.001
	Off-flavor	10	8.9	17	15.2	5	4.5	14	12.5	12	10.7	6	5.4	0.049
Texture/Mouthfeel	Hardness	74	66.1	11	9.8	20	17.9	18	16.1	21	18.8	8	7.1	<0.001
	Crispy	79	70.5	28	25.0	47	42.0	52	46.4	39	34.8	42	37.5	<0.001
	Stuffy	5	4.5	53	47.3	38	33.9	35	31.3	42	37.5	41	36.6	<0.001
	Oily	14	12.5	34	30.4	23	20.5	25	22.3	27	24.1	15	13.4	0.008
	Roughness	14	12.5	68	60.7	37	33.0	37	33.0	51	45.5	55	49.1	<0.001
	Residual sensation	16	14.3	5	4.5	16	14.3	21	18.8	15	13.4	8	7.1	0.012
	Crumble	19	17.0	46	41.1	44	39.3	50	44.6	49	43.8	48	42.9	<0.001
	Smooth	41	36.6	23	20.5	24	21.4	22	19.6	24	21.4	17	15.2	0.004

^(1)^ *p*-value by chi-square test, ^(2)^ *p*-value by Fisher’s exact test.

**Table 4:** Results of consumer acceptance test on six cookie samples (five plant-based, one animal-fat-based) using the 9-point hedonic scale ^(1)^.

	Butter (470)		Canola Oil (918)		Brown Rice Oil (613)		Coconut Oil (762)		Soybean Oil (396)		Olive Oil (284)		*p*-Value ^(2)^
	Mean	S.D.	Mean	S.D.	Mean	S.D.	Mean	S.D.	Mean	S.D.	Mean	S.D.	
Overall liking	6.38 ^a^	1.93	4.98 ^c^	1.63	5.89 ^b^	1.58	6.63 ^a^	1.85	5.29 ^c^	1.70	6.19 ^ab^	1.47	<0.001
Appearance	6.70 ^a^	1.58	5.90 ^b^	1.38	6.03 ^b^	1.26	5.95 ^b^	1.41	5.88 ^b^	1.37	5.90 ^b^	1.19	<0.001
Color	6.83 ^a^	1.45	5.89 ^b^	1.35	6.12 ^b^	1.27	6.13 ^b^	1.37	5.91 ^b^	1.30	6.04 ^b^	1.31	<0.001
Aroma/smell	6.29 ^b^	1.83	5.37 ^c^	1.74	6.13 ^b^	1.49	6.95 ^a^	1.89	5.46 ^c^	1.71	6.49 ^b^	1.45	<0.001
Overall taste	6.38 ^ab^	1.80	5.19 ^c^	1.66	5.94 ^b^	1.50	6.79 ^a^	1.84	5.46 ^c^	1.66	6.37 ^ab^	1.32	<0.001
Savory flavor	6.20 ^bc^	1.82	6.04 ^bc^	1.80	6.36 ^b^	1.65	6.88 ^a^	1.53	5.85 ^c^	1.70	7.20 ^a^	1.15	<0.001
Flavor	6.04 ^bc^	1.94	5.59 ^c^	1.68	6.15 ^b^	1.57	7.05 ^a^	1.63	5.61 ^c^	1.84	6.37 ^b^	1.53	<0.001
Sweet taste	6.23 ^a^	1.75	5.38 ^b^	1.62	6.21 ^a^	1.59	6.55 ^a^	1.69	5.62 ^b^	1.70	6.31 ^a^	1.61	<0.001
Aftertaste	6.12 ^ab^	1.96	4.86 ^c^	1.75	5.62 ^b^	1.76	6.18 ^a^	2.01	5.13 ^c^	1.76	5.76 ^ab^	1.75	<0.001
Texture	6.86 ^a^	1.59	5.26 ^d^	1.56	6.01 ^bc^	1.47	6.32 ^b^	1.64	5.62 ^cd^	1.52	5.98 ^bc^	1.48	<0.001
Smooth	5.56 ^bc^	1.84	5.46 ^c^	1.74	5.96 ^ab^	1.52	6.13 ^a^	1.63	5.58 ^ab^	1.70	5.77 ^abc^	1.66	0.023
Crispy	7.54 ^a^	1.44	5.50 ^d^	1.51	6.04 ^b^	1.58	6.08 ^b^	1.55	5.54 ^cd^	1.67	5.95 ^bc^	1.62	<0.001

^(1)^ 9-point hedonic scale: 1 = Dislike extremely, 5 = Neither like nor dislike, 9 = Like extremely; ^(2)^ *p*-value by One-way ANOVA; ^a–e^ Indicates significant differences between the samples for each consumer acceptance.

**Table 5:** Results for purchase intention for the six cookie samples.

	Butter (470)		Canola Oil (918)		Brown Rice Oil (613)		Coconut Oil (762)		Soybean Oil (396)		Olive Oil (284)		*p*-Value ^(1)^
	Mean	S.D.	Mean	S.D.	Mean	S.D.	Mean	S.D.	Mean	S.D.	Mean	S.D.	
Try again	6.27 ^ab^	2.22	4.64 ^e^	1.89	5.62 ^cd^	1.83	6.51 ^a^	2.09	5.10 ^de^	1.92	5.88 ^bc^	1.92	<0.001
Recommendation	6.05 ^ab^	2.16	4.48 ^e^	1.96	5.46 ^cd^	1.82	6.39 ^a^	2.04	5.02 ^d^	2.00	5.63 ^bc^	1.83	<0.001
Familiarity	6.39 ^ab^	2.14	4.71 ^d^	1.82	5.82 ^c^	1.96	6.71 ^a^	1.99	4.96 ^d^	1.98	6.10 ^bc^	1.87	<0.001

^(1)^ *p*-value by One-way ANOVA; ^a–e^ Indicates significant differences between the samples for each consumer acceptance.

## Data Availability

The original contributions presented in this study are included in the article/Supplementary Materials. Further inquiries can be directed to the corresponding author.

## Supplementary Materials

The following supporting information can be downloaded at: https://www.mdpi.com/article/10.3390/foods13223593/s1, Table S1. The Definition of CATA attributes from cookies using planted-based oils
