# Supplementary material for: What Is the Relationship Between Sensory Attributes Identified Using CATA (Check-All-That-Apply) Questionnaire and Consumer Acceptance of Cookies Using Plant-Based Oils?
Source: Foods. 2024 Nov 10;13(22):3593. doi: 10.3390/foods13223593 (PMC11593526; doi:10.3390/foods13223593)
Supplement: Supplementary file 1 [file foods-13-03593-s001.zip › foods-3277450-supplementary.pdf]

Table S1. The Definition of CATA attributes from cookies using planted-based oils

**Attribute**		**Definition**
Appearance	Color	Presence or absence of Yellowness
	Glossy	Presence or absence of shiny or glossiness
	Uniformity	Presence or absence of shape uniformity
Aroma/Odor	Milk	Presence or absence of milk aroma
	Savory	Presence or absence of salty and rich smell
	Rancid odor	Presence or absence of rancid oil smell
	Sweet	Presence or absence of sweet smell
	Off-odor	Presence or absence of off-odor
Taste/Flavor	Savory	Presence or absence of salty and rich taste
	Greasy	Presence or absence of oily and cheesy taste
	Sweet	Presence or absence of sweet taste
	Salty	Presence or absence of salty taste
	Bitter	Presence or absence of bitter taste
	Oily	Presence or absence of oil taste
	Nutty	Presence or absence of nut taste
	Raw flavor	Presence or absence of raw starch flavor
	Roasted flour	Presence or absence of roasted flour flavor
	Roasted grain	Presence or absence of roasted grain flavor
	Milk	Presence or absence of milk taste
	Off-flavor	Presence or absence of off-flavor
Texture/Mouthfeel	Hardness	Presence or absence of force for achievement a certain deformation
	Crispy	Presence or absence of hard enough to be broken easily
	Stuffy	Presence or absence of crumbly texture
	Oily	Presence or absence of oily mouthfeel
	Roughness	Presence or absence of not smooth surface of cookie
	Residual sensation	Presence or absence of remaining after eating cookie
	Crumble	Presence or absence of mouthfeel of being broken into small pieces
	Smooth	Presence or absence of smooth texture
